# A U-Shaped Relationship Between Blood Manganese Levels and Anemia in Patients With CKD: A Cross-Sectional Analysis from National Health and Nutrition Examination Survey 2015 to 2018

**DOI:** 10.1016/j.xkme.2025.101050

**Published:** 2025-06-17

**Authors:** Chunjie Jiang, Junlin Yi, Jiahui Lai, Luona Wen, Xiaoshi Zhong, Rongshao Tan, Yun Liu

**Affiliations:** 1 Department of Clinical Nutrition, Guangzhou Red Cross Hospital, Jinan University, Guangzhou, Guangdong Province, China; 2 Department of Cardiovascular Medicine, Hunan University of Medicine General Hospital, Huaihua, China; 3 Department of Nephrology, the Third Affiliated Hospital of Sun Yat-Sen University, Guangzhou, Guangdong, China; 4 Department of Nephrology, Guangzhou Red Cross Hospital, Jinan University, Guangzhou, Guangdong Province, China

**Keywords:** Blood manganese, chronic kidney disease, anemia, NHANES, U-shaped relationship

## Abstract

**Rationale & Objective:**

A complex relationship exists between blood manganese (Mn) and hemoglobin concentrations in patients with chronic kidney disease (CKD), with associations observed in patients not treated with dialysis and those on maintenance hemodialysis. This study aimed to elucidate this relationship using a large sample of adult patients with CKD from the National Health and Nutrition Examination Survey (NHANES) database.

**Study Design:**

This was a across-sectional study.

**Setting & Study Populations:**

We included data of 1,016 adult patients with CKD from the NHANES database between 2015 and 2018.

**Exposure:**

We included participants with CKD who were aged ≥18 years, not pregnant, and had available data on hemoglobin, Mn levels, and other relevant covariates.

**Outcomes:**

Hemoglobin and blood Mn levels in patients with CKD.

**Analytical Approach:**

Whole-blood Mn concentrations were analyzed using quadrupole inductively coupled plasma mass spectrometry. The statistical analyses included univariate and multivariate linear and binary logistic regression models, along with generalized additive models and smooth curve fitting to explore nonlinearity, which was further examined using a 2-piece-wise linear regression model.

**Results:**

After adjusting for age; sex; race/ethnicity; body mass index; smoking status; and levels of albumin, creatinine, ferritin, and transferrin receptor, a nonlinear (U-shaped) association was observed between Mn levels and anemia risk (*P* < 0.001 for nonlinearity). Specifically, lower Mn levels (<194.2 nmol/L) were negatively associated with anemia (OR, 0.984; 95% CI, 0.979-0.990; *P* < 0.001), whereas higher Mn levels (>194.2 μmol/L) showed a positive association (OR, 1.006; 95% CI, 1.001-1.011; *P* = 0.021).

**Limitations:**

Even with multivariate model analysis, we failed to establish a causal relationship between Mn levels and anemia in patients with CKD.

**Conclusions:**

These findings suggest that Mn may have a dual role in the pathophysiology of anemia in patients with CKD.

## Plain-Language Summary

Research has shown that there is a complex relationship between blood manganese (Mn) levels and hemoglobin concentration in patients with CKD, but existing studies are inconsistent. It is necessary to further clarify this relationship using a larger database. We collected data on adult patients with CKD from the National Health and Nutrition Examination Survey (NHANES) database from 2015-2018. We used univariate and multivariate linear and binary logistic regression models, as well as generalized additive models and smooth curve fitting, to explore nonlinearity, and further tested it using a two-stage linear regression model. After adjusting for a range of potential confounding factors, the results showed a nonlinear (U-shaped) association between manganese levels and anemia risk in patients with CKD.

## Introduction

Manganese (Mn) is a vital trace element that plays a crucial role in human health. It serves as a cofactor for various enzymes and plays a significant role in protecting organisms from oxidative stress.^1^ As a cofactor for glycosyltransferases, Mn is involved in diverse metabolic processes, such as glucose and lipid metabolism, modulation of enzyme activity, cell differentiation, organism development, and bone formation.^2^ Manganese deficiency can cause developmental delay, intellectual disability, failure to thrive, seizures, dystonia, and deafness.^3^^,^^4^ Insufficient Mn negatively affects health, whereas excess Mn is toxic, causing mitochondrial damage and subsequent energy failure.^5^ In addition, elevated blood and urinary Mn concentrations have been associated with poor cognitive function in older adults.^6^

Chronic kidney disease (CKD) encompasses a spectrum of conditions that affect the structure and function of the kidneys and poses a significant threat to ∼10% of the global adult population.^7^ The incidence of CKD is increasing, leading to a substantial economic burden on both individuals and the society.^8^ Patients with CKD primarily exhibit dysregulated mineral metabolism, anemia, insulin resistance, and subclinical hypothyroidism.^9^ The CKD stages have been significantly associated with the prevalence of anemia, supporting the need for more frequent testing in individuals with advanced CKD. Analysis of cross-sectional data from the National Health and Nutrition Examination Survey (NHANES) III showed that the overall prevalence of anemia in individuals with CKD was 7.6%, which increased with disease stage from 8.4% at stage G1 to 53.4% at stage G5.^10^ Although supplementation with erythropoiesis-stimulating agents, iron preparations, management of chronic inflammation, utilization of hypoxia-inducible factor-prolyl hydroxylase inhibitors, and improvement of nutritional status are widely performed in clinical practice, these measures fail to completely resolve anemia in this patient population.^11^ Furthermore, CKD exacerbates disturbances in mineral and trace element metabolism.^12^ The imbalance of certain trace elements in patients with CKD may be closely linked to anemia, an issue that has not yet been fully explored.

Mn metabolism has been reported impaired in patients with CKD. Studies have indicated that patients with CKD, regardless of whether they undergo renal replacement therapy, are more likely to exhibit low serum or blood Mn levels.^13^ This phenomenon may be associated with strict dietary restrictions imposed on patients and diminished intestinal absorption capacity because of uremic toxin accumulation. Conversely, several studies have reported elevated Mn levels in the hair of patients undergoing hemodialysis compared with those in healthy controls, along with Mn deposition in the basal ganglia of the brain in this population.^14^^,^^15^ Anemia in patients with CKD is associated with increased risks of cardiovascular events and all-cause mortality.^16^ However, other studies have also reported interesting results. Kim et al^17^ discovered a positive correlation between blood Mn and hemoglobin (Hb) levels in a cohort of 334 patients with CKD who did not receive dialysis treatment. Another recent case-control study showed a significant association between serum Mn concentration and CKD risk.^18^ Our previous investigation showed an independent association between elevated blood Mn and Hb levels in patients with CKD undergoing maintenance hemodialysis (n = 144).^19^ However, considering the complexity of Mn, further studies with larger sample sizes are warranted.

Here, we aimed to further investigate the relationship between blood Mn levels and anemia in adult patients with CKD, using data from the NHANES database collected from 2015-2018.

## Materials and Methods

### Study Design and Population

The NHANES, conducted by the Centers for Disease Control and Prevention, is an ongoing cross-sectional survey representative of the general population of the United States. The survey is conducted using a stratified multistage probability cluster design, and data are collected through interviews, physical examinations, and laboratory tests. The NHANES protocols were approved by the institutional review board of the National Center of Health Statistics, and all participants provided written informed consent on enrollment.

**Figure 1:**
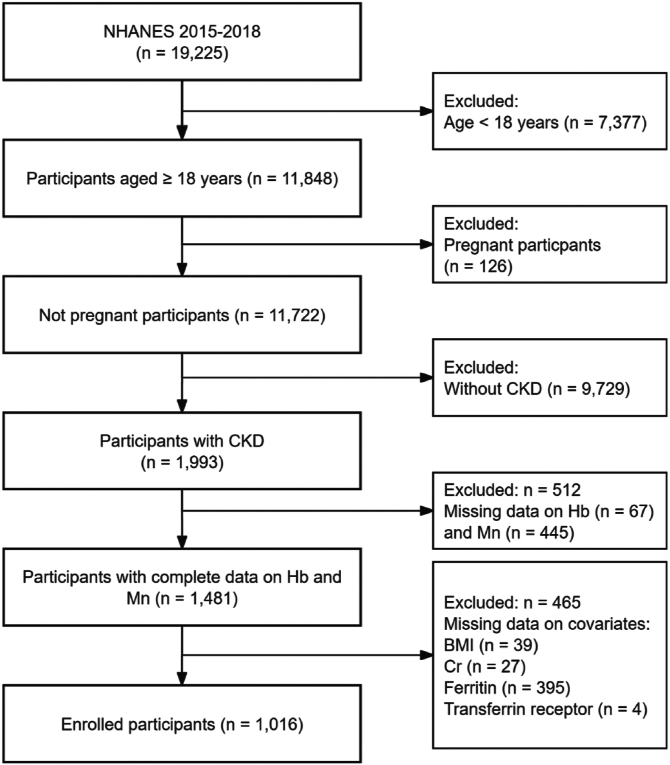
Flowchart of participants included in this study. NHANES, National Health and Nutrition Examination Survey; BMI, body mass index; CKD, chronic kidney disease; Cr, serum creatinine; Hb, hemoglobin; Mn, manganese.

Our study included participants from the 2015-2016 and 2017-2018 cycles (n = 19,225). We excluded participants who were aged younger than 18 years (n = 7,377), were pregnant (n = 126), did not have CKD (n = 9,729), had missing data on Hb (n = 67), Mn (n = 445) levels, or other relevant covariates (n = 465). Ultimately, 1,016 participants were included in the statistical analysis (Fig 1).

### Laboratory Measurements

Whole-blood specimens were processed, frozen at −30 °C, and shipped to the National Center for Environment and Health for analysis. Blood Mn levels were measured using inductively coupled plasma mass spectrometry. The lower limit of detection for blood Mn levels was 0.99 μg/L. Complete blood cell counts were determined using a Beckman Coulter MAXM analyzer. The Hb levels were measured using a photometric method. A detailed description of the laboratory methods can be found on the NHANES website.

### Definition of Anemia

Anemia was defined according to the World Health Organization criteria as an Hb level < 13 g/dL or hematocrit (HCT) < 39% for men and Hb level < 12 g/dL or HCT < 36% for women.

### CKD Diagnosis

The Modification of Diet in Renal Disease Study Equation was used in this study to calculate the estimated glomerular filtration rate (eGFR). The CKD was defined as kidney damage indicated by eGFR < 60 mL/min/1.73 m^2^ or an albumin-to-creatinine ratio ≥30 mg/g. The CKD Epidemiology Collaboration equation was used to calculate the eGFR from serum creatinine measurements. The serum creatinine levels were determined using the Jaffé rate reaction. Urinary albumin and creatinine levels were assessed using a solid-phase fluorescence immunoassay in conjunction with the Jaffé rate reaction.

### Covariates

Demographic variables included age, sex, race/ethnicity, body mass index (BMI), smoking status, and presence of comorbid conditions. The BMI was calculated as weight (kg) divided by height (m^2^). Smoking status was categorized as never, former, or current, based on self-reports. Participants who reported consuming ≥100 cigarettes in their lifetime were defined as smokers, and those who reported smoking at the time of the interview were classified as current smokers. Alcohol consumption status was categorized as never, former drinker, mild drinker, moderate drinker, and heavy drinker. Socioeconomic status was classified using the ratio of family income to poverty (poverty index ratio [PIR]), with participants categorized as poor (PIR ≤1.3), near poor (1.3 < PIR < 3.5), and nonpoor (PIR ≥3.5). Participants were considered to have hypertension if they met any of the following criteria: systolic blood pressure ≥140 mm Hg, diastolic blood pressure ≥90 mm Hg, use of hypertensive medications, or self-reported diagnosis. Similarly, participants were considered to have diabetes if they met any of the following criteria: fasting glucose ≥126 mg/dL, glycated Hb ≥6.5%, use of antidiabetic drugs, or self-reported diagnosis. The diagnosis of chronic obstructive pulmonary disease was based on self-report. In addition, albumin, serum iron (Fe), serum ferritin, and transferrin receptor (TFR) levels were measured.

### Statistical Analysis

Continuous variables are expressed as mean (standard deviation [SD]) or median (interquartile range [IQR]), depending on their distribution. Categorical variables are presented as frequencies and percentages.

Linear regression models were used to assess the association between the blood Mn and Hb levels. Multivariate binary logistic regression models were used to examine the association between blood Mn levels and anemia. The results were reported as β coefficients (β) with 95% confidence intervals (CIs) for linear regression models and odds ratios (ORs) with 95% CI for logistic regression models. The selection of adjusted variables was based on *P* values < 0.5 in the univariate analysis, or changes in the effect estimates > 10% when the covariates were included in the multivariable models (Table S1). Three regression models were constructed: a crude model (no covariates adjusted), model I (adjusted for age, sex, ethnicity, BMI, and smoking), and model II (adjusted for age, sex, ethnicity, BMI, smoking, albumin, creatinine, ferritin, and TFR).

To further investigate the nonlinear relationship between blood Mn and Hb levels, as well as anemia, a restricted cubic spline function with 4 knots (5th, 25th, 75th, and 95th) was used. Nonlinearity was tested using a likelihood ratio test. When nonlinearity was detected, the inflection point was calculated using a recursive algorithm and fitting 2 regression lines on either side.

All analyses were performed using R software (version 4.1.2). Statistically significance was set at *P* < 0.05 (2-sided).

## Results

### Baseline Characteristics

Table 1 outlines the baseline characteristics of the participants stratified according to their anemia status. The mean level of Mn in patients with anemia was 169.55 μmol/L, which is lower than that of nonanemia patients (178.6 μmol/L). Compared with 780 patients without anemia, the 215 patients with anemia were older and presented lower levels of mean corpuscular volume, mean corpuscular hemoglobin, mean corpuscular hemoglobin concentration, red cell distribution width, HCT, Fe, albumin, alanine aminotransferase, bilirubin, and eGFR. However, they exhibited higher levels of TFR, creatinine, blood urea nitrogen, urine albumin creatine ratio, hypersensitive C-reactive protein, and phosphorus, and a higher prevalence of diabetes and hypertension. In addition, there were differences in the racial composition and smoking status between the 2 groups.

**Table 1:** Baseline Characteristics of Participants Stratified with Anemia

Characteristic	Variable	Overall	Patients Without Anemia	Patients With Anemia	*P*
No. of participants		995	780	215	
Mn (umol/L)		176.65 (67.19)	178.60 (61.04)	169.55 (85.69)	0.08
Mn (μg/L)		9.70 (3.69)	9.81 (3.35)	9.32 (4.71)	0.08
Age		61.10 (17.39)	60.11 (17.51)	64.67 (16.51)	0.001
Sex (%)	Male	457 (45.9)	354 (45.4)	103 (47.9)	0.562
	Female	538 (54.1)	426 (54.6)	112 (52.1)	
Ethnicity (%)	Non-Hispanic White	405 (40.7)	335 (42.9)	70 (32.6)	<0.001
	Mexican American	117 (11.8)	95 (12.2)	22 (10.2)	
	Others	244 (24.5)	203 (26.0)	41 (19.1)	
	Non-Hispanic African American	229 (23.0)	147 (18.8)	82 (38.1)	
Poverty		2.38 (1.53)	2.42 (1.55)	2.23 (1.43)	0.122
BMI (kg/m^2^)		30.76 (7.75)	30.75 (7.76)	30.80 (7.74)	0.937
Smoking status (%)	Never	537 (54.0)	423 (54.2)	114 (53.0)	0.034
	Former	315 (31.7)	235 (30.1)	80 (37.2)	
	Current	143 (14.4)	122 (15.6)	21 (9.8)	
Alcohol user (%)	Former	2 (0.3)	2 (0.4)	0 (0.0)	0.122
	Heavy	91 (14.3)	77 (14.8)	14 (12.0)	
	Mild	311 (48.9)	248 (47.8)	63 (53.8)	
	Moderate	115 (18.1)	102 (19.7)	13 (11.1)	
	Never	117 (18.4)	90 (17.3)	27 (23.1)	
Hb (g/dL)		13.53 (1.71)	14.14 (1.29)	11.33 (1.18)	<0.001
MCV (fL)		88.71 (6.76)	89.38 (5.55)	86.29 (9.62)	<0.001
MCH (pg)		29.62 (2.70)	29.99 (2.16)	28.28 (3.83)	<0.001
RDW		14.25 (1.55)	13.93 (1.12)	15.40 (2.21)	<0.001
Anemia (%)	Nonanemia	780 (78.4)	780 (100.0)	0 (0.0)	<0.001
	Yes	215 (21.6)	0 (0.0)	215 (100.0)	
HCT		40.49 (4.70)	42.12 (3.63)	34.60 (3.17)	<0.001
FER (median [IQR])		113.00 (52.95-218.00)	121.00 (57.98-223.50)	92.30 (28.50-209.00)	<0.001
TFR (nmol/L)		43.85 (24.62)	39.81 (13.80)	58.53 (42.96)	<0.001
Fe (μmol/L)		14.37 (5.73)	15.28 (5.54)	11.08 (5.16)	<0.001
Alb (g/L)		39.8 (0.37)	40.4 (0.34)	37.9 (0.40)	<0.001
Cr (μmol/L)		103.21 (84.23)	91.02 (51.50)	147.46 (144.19)	<0.001
BUN (mmol/L)		6.94 (3.44)	6.36 (2.63)	9.03 (4.94)	<0.001
UACR (mg/g)		235.04 (833.98)	182.81 (605.13)	434.88 (1383.14)	<0.001
Hs-CRP (mg/L)		5.71 (12.30)	4.99 (10.80)	8.34 (16.40)	<0.001
UA (μmol/L)		354.70 (100.95)	352.09 (99.15)	364.18 (106.94)	0.12
ALT(U/L)		21.08 (15.92)	22.59 (17.32)	15.60 (6.79)	<0.001
Bilirubin (μmol/L)		7.95 (4.51)	8.30 (4.53)	6.67 (4.19)	<0.001
LDH (U/L)		167.03 (43.74)	166.21 (43.31)	170.01 (45.23)	0.259
CKD (%)	Yes	995 (100.0)	780 (100.0)	215 (100.0)	
CKD_prognosis (%)	Low risk	0 (0.0)	0 (0.0)	0 (0.0)	<0.001
	Moderate risk	685 (70.1)	584 (75.9)	101 (48.6)	
	High risk	181 (18.5)	130 (16.9)	51 (24.5)	
	Very high risk	111 (11.4)	55 (7.2)	56 (26.9)	
eGFR (mL/min/1.73m^2^)		74.43 (31.25)	78.29 (29.34)	60.44 (33.93)	<0.001
Hypertension (%)	No	298 (29.9)	248 (31.8)	50 (23.3)	0.019
	Yes	697 (70.1)	532 (68.2)	165 (76.7)	
Diabetes (%)	No	580 (58.3)	472 (60.5)	108 (50.2)	0.009
	Diabetes	415 (41.7)	308 (39.5)	107 (49.8)	
COPD (%)	No	910 (93.5)	716 (94.1)	194 (91.5)	0.234
	Yes	63 (6.5)	45 (5.9)	18 (8.5)	
Pregnant (%)	No	114 (100.0)	85 (100.0)	29 (100.0)	NA

*Note:* Descriptive statistics represented as mean ± standard deviation. Categorical variables are expressed as percentages.

Abbreviations: Alb, albumin; ALT, alanine aminotransferase; BMI, body mass index; BUN, blood urea nitrogen; Cr, creatinine; CKD, chronic kidney disease; COPD, chronic obstructive pulmonary disease; eGFR: estimate glomerular filtration; Fe, iron; FER, ferritin; Hb, hemoglobin; HCT, hematocrit; Hs-CRP, hypersensitive C-reactive protein; IQR, interquartile range; LDH, lac**t**ate dehydrogenase; Mn, manganese; MCV, mean corpuscular volume; MCH, mean corpuscular hemoglobin; MCHC, mean corpuscular hemoglobin concentration; RDW, red cell distribution width; TFR, transferrin receptors; UA, uric acid; UACR, urine albumin creatine ratio.

### Blood Mn and Hb Levels

**Figure 2:**
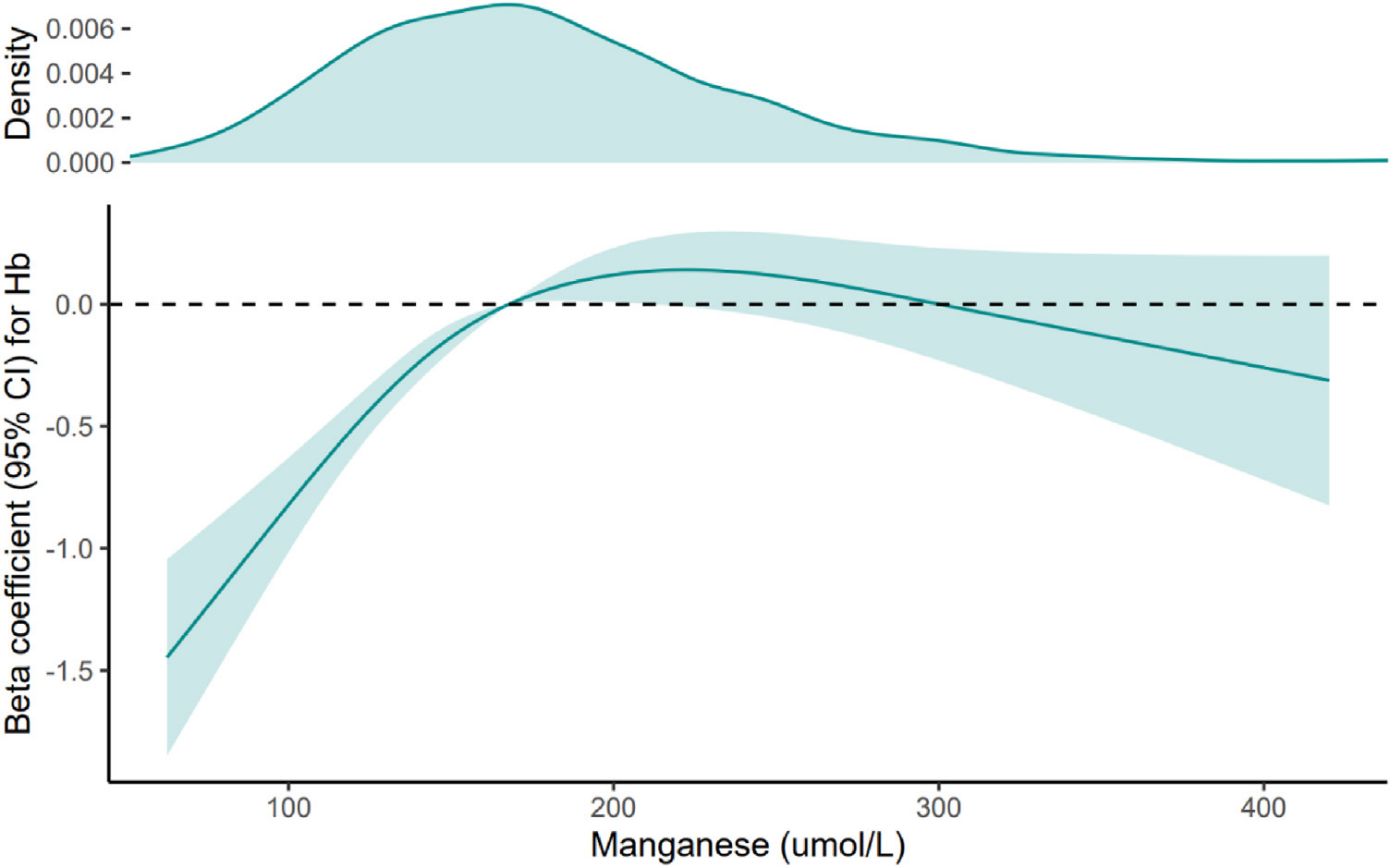
Nonlinear relationship between Mn levels and Hb in adults with CKD. The odds ratios (solid lines) and 95% CIs (shaded areas) were adjusted for age, sex, race/ethnicity, BMI, smoking, hypertension, diabetes, albumin, ALT, total bilirubin, LDH, eGFR, ferritin, and TFR. BMI, body mass index; TFR, transferrin receptor; CKD, chronic kidney disease; eGFR, estimated glomerular filtration rate; CI, confidence interval; Mn, manganese; Hb, hemoglobin.

A nonlinear association was observed between blood Mn and Hb levels (*P* < 0.001 for nonlinearity) (Fig 2). After multivariable adjustment, compared with the first quartile, the β coefficients for the second, third, and fourth quartiles were 0.51 (95% CI, 0.06-0.96), 0.81 (95% CI, 0.51-1.11), and 0.77 (95% CI, 0.40-1.14), respectively (*P* for trend < 0.001) (Table 2).

**Table 2:** Relationship Between Blood Manganese and Hemoglobin Levels

Hemoglobin	Crude Model		Model I		Model II	
	β (95% CI)	*P*	β (95% CI)	*P*	β (95% CI)	*P*
**Blood manganese (nmol/L)**	0.00 (−0.00 to 0.00)	0.62	−0.00 (−0.00 to 0.00)	0.47	0.00 (0.00-0.01)	0.007
**Blood manganese (nmol/L) (quartile)**						
Q1	Reference		Reference		Reference	
Q2	0.59 (0.12-1.07)	0.02	0.64 (0.17-1.12)	0.01	0.51 (0.06-0.96)	0.03
Q3	0.70 (0.36-1.04)	< 0.001	0.82 (0.46-1.18)	< 0.001	0.81 (0.51-1.11)	<0.001
Q4	0.47 (0.13-0.80)	0.008	0.55 (0.20-0.90)	0.004	0.77 (0.40-1.14)	0.003
**P for trend**	0.007		0.002		<0.001	

*Note:* Crude model: no covariates were adjusted. Model I was adjusted for age, sex, race/ethnicity, BMI, smoking, hypertension and diabetes. Model II was adjusted for age, sex, race/ethnicity, BMI, smoking, hypertension, diabetes, albumin, ALT, total bilirubin, LDH, eGFR, ferritin, and TFR.

Abbreviations: BMI, body mass index; CI, confidence interval; Mn, manganese.

**Figure 3:**
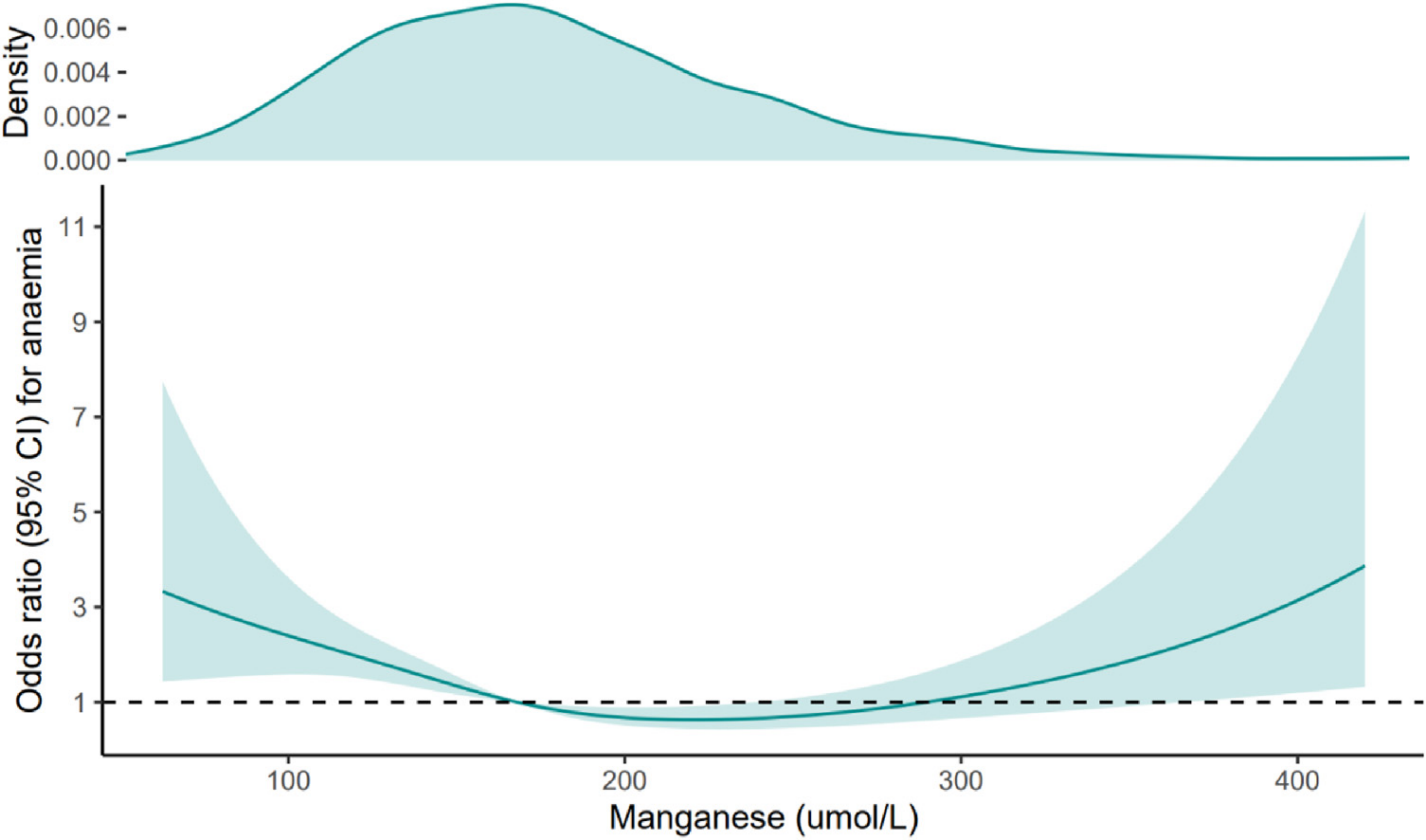
Nonlinear relationship between Mn levels and anemia in adults with CKD.Odds ratios (solid lines) and 95% CIs (shaded areas) were adjusted for age, sex, race/ethnicity, BMI, smoking, hypertension, diabetes, albumin, ALT, total bilirubin, LDH, eGFR, ferritin, and TFR. BMI, body mass index; TFR, transferrin receptor; eGFR, estimated glomerular filtration rate; CI, confidence interval.

A U-shaped association was found between blood Mn levels and the risk of anemia (*P* < 0.001 for nonlinearity) (Fig 3). After multivariable adjustment, compared with the first quartile, the ORs for anemia was 0.80 (95% CI, 0.30-2.09) for the second quartile, 0.38 (95% CI, 0.15-0.99) for the third quartile, and 0.51 (95% CI, 0.20-1.26) for the fourth quartile (*P* for trend = 0.054) (Table 3).

**Table 3:** Relationship Between Blood Manganese and Anemia

Anemia	Crude Model		Model I		Model II	
	OR (95% CI)	*P*	OR (95% CI)	*P*	OR (95% CI)	*P*
**Blood manganese (nmol/L)**	1.00 (1.00-1.00)	0.44	1.00 (1.00-1.00)	0.453	1.00 (0.99-1.00)	0.286
**Blood manganese (nmol/L) (quartile)**						
Q1	Reference		Reference		Reference	
Q2	0.56 (0.29-1.08)	0.08	0.61 (0.29-1.32)	0.19	0.80 (0.30-2.09)	0.57
Q3	0.37 (0.20-0.69)	0.003	0.44 (0.21-0.88)	0.02	0.38 (0.15-0.99)	0.05
Q4	0.54 (0.33-0.88)	0.02	0.73 (0.43-1.24)	0.22	0.51 (0.20-1.26)	0.12
**P for trend**	0.007		0.12		0.05	

*Note:* Crude model: no covariates were adjusted. Model I was adjusted for age, sex, race/ethnicity, BMI and smoking. Model II was adjusted for age, sex, race/ethnicity, BMI, smoking, hypertension, diabetes, albumin, ALT, total bilirubin, LDH, eGFR, ferritin, and TFR.

Abbreviations: BMI, body mass index; CI, confidence interval; TFR, transferrin receptors.

### Two Piece-Wise Regressions

As shown in Table 4, a saturation effect of blood Mn on Hb levels was observed. When blood Mn levels was ≤171.64 μmol/L, there was a positive association with Hb levels (β = 0.014; 95% CI, 0.011-0.017; *P* < 0.001), whereas for Mn levels >171.64 umol/L, the association was not significant (β = −0.001; 95% CI, −0.003 to 0.001; *P* = 0.251) in model II.

**Table 4:** Two Piece-Wise Linear Regression Between Blood Manganese and Hemoglobin

	Model		β, 95% CI, *P* value	Log likelihood ratio test *P* value
Crude Model	One-line		0.001 (−0.001 to 0.003), 0.187	
	Two-line	≤164.55	0.017 (0.012-0.021), <0.001	<0.001^a^
		>164.55	−0.005 (−0.007 to −0.003), <0.001	
Model I	One-line		0.001 (−0.000 to 0.003), 0.073	
	Two-line	≤169.64	0.016 (0.013-0.020), <0.001	<0.001^a^
		>169.64	−0.005 (−0.007 to −0.003), <0.001	
Model II	One-line		0.004 (0.002-0.005), <0.001	
	Two-line	≤171.64	0.014 (0.011-0.017), <0.001	<0.001^a^
		>171.64	−0.001 (−0.003 to 0.001), 0.251	

*Note:* Crude model: no covariates were adjusted. Model I was adjusted for age, sex, race/ethnicity, BMI, and smoking. Model II was adjusted for age, sex, race/ethnicity, BMI, smoking, hypertension, diabetes, albumin, ALT, total bilirubin, LDH, eGFR, ferritin, and TFR.

Abbreviations: BMI, body mass index; CI, confidence interval; TFR, transferrin receptors.

a Statistically significant *P*-values indicating that the two-piece linear model provides a significantly better fit than the one-line model.

Similarly, a threshold effect was observed in the relationship between blood Mn levels and anemia (Table 5). When blood Mn levels were ≤268.12 μmol/L, there was a negative association with anemia (OR, 0.992; 95% CI, 0.988-0.996; *P* < 0.001). In Mn levels >268.12 μmol/L, the association became positive (OR, 1.014; 95% CI, 1.006-1.021; *P* < 0.001) in model II.

**Table 5:** Two Piece-Wise Linear Regression Between Blood Manganese and the Prevalence of Anemia

	Model		OR, 95% CI, *P*	Log likelihood ratio test *P*
Crude Model	One-line		0.998 (0.995-1.000), 0.081	
	Two-line	≤196.76	0.985 (0.981-0.989), <0.001	<0.001^a^
		>196.76	1.009 (1.005-1.013), <0.001	
Model I	One-line		1.000 (0.998-1.003), 0.838	
	Two-line	≤196.22	0.987 (0.983-0.992), <0.001	<0.001^a^
		>196.22	1.010 (1.007-1.014), <0.001	
Model II	One-line		0.997 (0.994-1.001), 0.111	
	Two-line	≤268.12	0.992 (0.988-0.996), <0.001	<0.001^a^
		>268.12	1.014 (1.006-1.021), <0.001	

*Note:* Crude model: no covariates were adjusted. Model I was adjusted for age, sex, race/ethnicity, BMI, and smoking. Model II was adjusted for age, sex, race/ethnicity, BMI, smoking, hypertension, diabetes, albumin, ALT, total bilirubin, LDH, eGFR, ferritin, and TFR.

Abbreviations: BMI, body mass index; CI, confidence interval; TFR, transferrin receptors.

a Statistically significant *P*-values indicating that the two-piece linear model provides a significantly better fit than the one-line model.

## Discussion

Anemia is a prevalent complication of CKD, and emerging research suggests that imbalances in trace element levels may affect Hb metabolism. Our previous investigation indicated that elevated blood Mn levels were independently associated with high Hb levels in individuals undergoing maintenance hemodialysis.^19^ In this study, we observed a 21.3% incidence of CKD-related anemia. The relationship between blood Mn and Hb levels, and the associated risk of anemia, displayed a nonlinear pattern. Piece-wise linear regression analysis showed distinct correlations: at blood Mn concentrations < 194.22 nmol/L, a negative association with anemia was evident, whereas concentrations above this threshold showed a positive correlation.

Notably, Mn metabolism is disrupted in CKD, making patients with CKD susceptible to changes in trace element levels. In certain long-lived areas of China, a negative association between plasma Mn levels and CKD progression after accounting for covariates was observed in older adults.^20^ In contrast, plasma Mn levels were positively correlated with creatinine, plasma urea, and plasma uric acid levels and negatively correlated with eGFR in patients with chronic kidney failure undergoing predialysis.^21^ In addition, elevated Mn levels and deposition have been observed in patients undergoing hemodialysis compared with healthy controls.^12^^,^^13^ It appears that Mn levels are not constant in patients with CKD, regardless of whether they undergo renal replacement therapy. It was reported that the blood Mn range in a healthy population is 5.9-13.3 μg/L (107.4-242.1 nmol/L).^22^ Although the blood Mn levels in a healthy population vary among countries and regions, most individuals fall within this range. In this study, the blood Mn levels of patients with CKD ranged from 1.57-31.37μg/L (28.6-571 nmol/L). Thus, some patients with CKD have low blood Mn levels, whereas others have high blood Mn levels.

Our findings suggest that blood Mn levels < 194.22 nmol/L are inversely associated with the incidence of anemia. Manganese is an essential inorganic trace element that plays a crucial role in the regulation of Hb levels. These findings suggest that Mn enhances Hb and red blood cell production. Previous studies have shown that the depletion of Mn superoxide dismutase disrupts Fe homeostasis, globin switching, and epigenetic control of erythrocyte precursor cells.^23^ In addition, Mn is a cofactor for Mn superoxide dismutase, an antioxidant enzyme responsible for regulating superoxide levels in the mitochondrial matrix and protecting against oxidative damage.^24^ Oxidative stress is known to be associated with decreased Hb levels.^25^^,^^26^ Notably, a study has shown that treatment with MnCl2 at concentrations ranging from 50-600 μM resulted in increased erythropoietin synthesis in Hep3B cells.^27^ Consequently, Mn deficiency may contribute to the development of anemia via various pathways. Mn is an essential micronutrient in the human body. Recently, substantial evidence has indicated that micronutrient deficiency in the diet is prevalent worldwide, regardless of the modality of CKD treatment or customary food choices. In addition, micronutrients are lost with urination because of the use of diuretics.^13^ Thus, Mn deficiency in patients with CKD may be a risk factor for anemia.

Although Mn is an essential trace element, the body requires only a small amount, and excessive intake can easily lead to Mn toxicity. In this study, when blood Mn levels exceed 194.22 nmol/L, a considerable association was observed between elevated Mn levels and anemia in patients with CKD. Previous studies demonstrated that excess Mn triggers endoplasmic reticulum stress, thereby inducing anemia through hepicidin activation.^28^^,^^29^ In addition, Mn is localized within the mitochondria, and excess Mn can cause cytotoxicity by inhibiting complexes I and II of the electron transport chain, leading to increased reactive oxygen species production.^30^ Reactive oxygen species has been identified as significant contributors to anemia.^31^ Thus, excess Mn in patients with CKD is a risk factor for anemia.

The mechanism underlying excess Mn in patients with CKD remains unclear. As the glomerular filtration rate decreases, the ability of the kidneys to excrete metabolic waste also decreases significantly. This may have impaired the Mn excretion. In addition, there is a significant correlation between Mn and Fe metabolism. Extensive research has consistently shown that inadequate Fe levels lead to elevated blood Mn levels in the general population.^32^ Moreover, Mn and Fe manifest an antagonistic relationship.^33^^,^^34^ Finley et al^35^ reported a strong association between serum ferritin concentration and Mn absorption from meals. A cross-sectional study showed an inverse relationship between serum ferritin and blood Mn levels.^36^ In addition, abnormal Mn accumulation has been observed in individuals with Fe-deficiency anemia and Fe-deficient infants, whereas blood Mn levels decreased and Hb levels increased after Fe therapy.^37^^,^^38^ However, the specific mechanism underlying excess Mn in CKD requires further investigation.

This study had several limitations. First, as a cross-sectional study, even with a multivariate model analysis, we were unable to establish a causal relationship between Mn levels and anemia in patients with CKD. Second, the study was conducted exclusively in the US population, encompassing various ethnicities. However, its applicability to nondialysis CKD populations in other countries is questionable. Third, the NHANES database lacks data on the use of erythropoietin and hepatobiliary imaging in patients, and there is a significant deficiency in occupational data, reaching 64%. In addition, it does not include variables related to occupation that may lead to Mn accumulation. Consequently, this limitation prevents a comprehensive analysis. In addition, Mn levels in the nails and other organs were not assessed, indicating that total blood Mn levels may not accurately reflect Mn metabolism and distribution in these patients.

In conclusion, our study demonstrated a U-shaped association between blood Mn levels and anemia in patients with CKD. Both Mn deficiency and excess were independently associated with anemia in this patient cohort. Although previous research has suggested the benefits of Mn in patients with CKD, the potential adverse effects on anemia warrant further consideration. Therefore, clinicians should carefully monitor Mn levels to avoid dysregulation in patients with CKD. Additional randomized controlled trials and mechanistic studies in patients with CKD are essential to confirm these findings.
