# Supplementary material for: A U-Shaped Relationship Between Blood Manganese Levels and Anemia in Patients With CKD: A Cross-Sectional Analysis from National Health and Nutrition Examination Survey 2015 to 2018
Source: Kidney Med. 2025 Jun 17;7(8):101050. doi: 10.1016/j.xkme.2025.101050 (PMC12304937; doi:10.1016/j.xkme.2025.101050)
Supplement: Supplementary File (PDF) — Table S1. [file mmc1.pdf]

Table S1. Univariate analysis for hemoglobin and anemia

Covariate	Statistics	Hemoglobin		Anemia	
		β (95%CI)	*P*-value	OR (95%CI)	*P*-value
Age	61.16 (17.41)	-0.003 [-0.012, 0.006]	0.452	1.011 [0.998, 1.024]	0.094
Sex					
Male	472 (46.5)	0		Ref.	
Female	544 (53.5)	-1.225 [-1.450, -0.999]	<0.001	0.955 [0.664, 1.374]	0.796
Ethnicity					
Non-Hispanic White	411 (40.5)	0		Ref.	
Non-Hispanic Black	236 (23.2)	-0.833 [-1.243, -0.423]	<0.001	2.510 [1.606, 3.923]	<0.001
Mexican American	120 (11.8)	0.204 [-0.245, 0.654]	0.356	1.028 [0.588, 1.798]	0.920
Other	249 (24.5)	0.159 [-0.136, 0.455]	0.276	0.838 [0.546, 1.287]	0.403
CKD prognosis					
Moderate risk	695 (69.6)	0		Ref.	
High risk	189 (18.9)	-0.444 [-0.793, -0.094]	0.015	2.149 [1.379, 3.349]	0.002
Very high risk	114 (11.4)	-1.209 [-1.713, -0.705]	<0.001	5.129 [3.005, 8.757]	<0.001
Smoking					
Never	546 (53.7)	0		Ref.	
Former	321 (31.6)	0.372 [-0.016, 0.759]	0.059	1.251 [0.749, 2.089]	0.375
Now	149 (14.7)	0.601 [0.170, 1.031]	0.008	0.630 [0.300, 1.321]	0.209
Hypertension					
No	304 (29.9)	0		Ref.	
Yes	712 (70.1)	0.039 [-0.316, 0.393]	0.823	1.238 [0.765, 2.004]	0.370
Diabetes					
No	594 (58.5)	0			
Yes	422 (41.5)	-0.011 [-0.203, 0.182]	0.909	1.627 [1.077, 2.459]	0.023

COPD					
No	928 (93.4)	0		Ref.	
Yes	66 (6.6)	-0.370 [-0.832, 0.093]	0.112	1.356 [0.704, 2.610]	0.347
BMI	30.75 (7.78)	0.007 [-0.008, 0.022]	0.364	0.989 [0.971, 1.007]	0.211
Iron	14.38 (5.70)	0.132 [0.114, 0.150]	<0.001	0.848 [0.820, 0.878]	<0.001
Fertin	194.15 (291.53)	0.000 [-0.000, 0.001]	0.153	1.000 [0.999, 1.001]	0.801
TFR	43.78 (24.44)	-0.025 [-0.030, -0.020]	<0.001	1.032 [1.022, 1.042]	<0.001
Albumin	39.8 (3.6)	1.428 [1.043, 1.813]	<0.001	0.148 [0.087, 0.251]	<0.001
RDW	14.25 (1.54)	-0.484 [-0.551, -0.417]	<0.001	1.865 [1.647, 2.110]	<0.001
Uric acid	354.69 (101.55)	0.002 [0.000, 0.003]	0.007	1.000 [0.998, 1.002]	0.945
BUN	6.94 (3.43)	-0.120 [-0.163, -0.077]	<0.001	1.202 [1.142, 1.264]	<0.001
Creatinine	103.28 (83.58)	-0.004 [-0.006, -0.002]	0.001	1.008 [1.002, 1.013]	0.007
ALT	21.08 (15.92)	0.024 [0.014, 0.035]	<0.001	0.937 [0.913, 0.962]	<0.001
Bilirubin	7.95 (4.51)	0.134 [0.097, 0.170]	<0.001	0.864 [0.802, 0.932]	0.001
LDH	167.03 (43.74)	0.002 [-0.001, 0.004]	0.263	1.001 [0.997, 1.005]	0.531
eGFR	74.43 (31.25)	0.007 [0.002, 0.013]	0.011	0.985 [0.977, 0.992]	<0.001

CKD, chronic kidney disease; COPD,chronic obstructive pulmonary disease; BMI, body mass index; TFR, transferrin receptor; RDW, red cell distribution width; BUN, blood urea nitrogen
